# Promising dawn in tumor microenvironment therapy: engineering oral bacteria

**DOI:** 10.1038/s41368-024-00282-3

**Published:** 2024-03-13

**Authors:** Zifei Wang, Wansu Sun, Ruixue Hua, Yuanyin Wang, Yang Li, Hengguo Zhang

**Affiliations:** 1 Key Laboratory of Oral Diseases Research of Anhui Province, College & Hospital of Stomatology, Anhui Medical University, Hefei, China; 2 Department of Stomatology, The First Affiliated Hospital of Anhui Medical University, Hefei, China; 3 Department of Genetics, School of Life Science, Anhui Medical University, Hefei, China

**Keywords:** Cancer immunotherapy, Bacterial techniques and applications

## Abstract

Despite decades of research, cancer continues to be a major global health concern. The human mouth appears to be a multiplicity of local environments communicating with other organs and causing diseases via microbes. Nowadays, the role of oral microbes in the development and progression of cancer has received increasing scrutiny. At the same time, bioengineering technology and nanotechnology is growing rapidly, in which the physiological activities of natural bacteria are modified to improve the therapeutic efficiency of cancers. These engineered bacteria were transformed to achieve directed genetic reprogramming, selective functional reorganization and precise control. In contrast to endotoxins produced by typical genetically modified bacteria, oral flora exhibits favorable biosafety characteristics. To outline the current cognitions upon oral microbes, engineered microbes and human cancers, related literatures were searched and reviewed based on the PubMed database. We focused on a number of oral microbes and related mechanisms associated with the tumor microenvironment, which involve in cancer occurrence and development. Whether engineering oral bacteria can be a possible application of cancer therapy is worth consideration. A deeper understanding of the relationship between engineered oral bacteria and cancer therapy may enhance our knowledge of tumor pathogenesis thus providing new insights and strategies for cancer prevention and treatment.

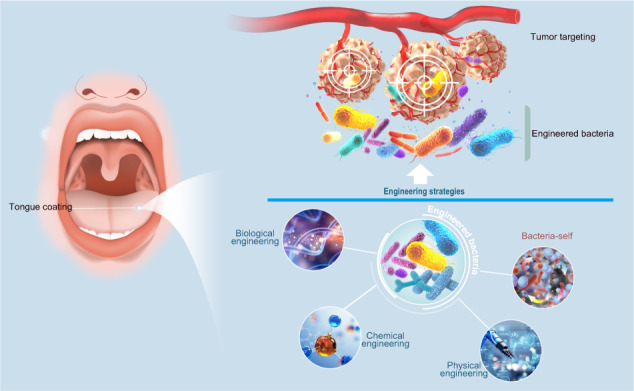

## Introduction

Cancer remains a major global health concern, and despite recent advances in prevention, detection, and treatment, it remains a leading cause of death worldwide, accounting for an estimated 10 million deaths in 2020.^1^ Studies have shown that the occurrence of many cancers can be attributed to random DNA mutations in highly dividing cell populations.^2^ The classic cancer-associated environmental risk factors are tobacco smoking, alcohol consumption, high body mass index (BMI), and exposure to ultraviolet radiation.^3,4^ Traditional cancer treatment methods, including surgery, chemotherapy, and radiation therapy, achieved certain therapeutic effects. However, these methods go along with significant limitations.^5^ For instance, surgical operation might end up with a high recurrence rate and poor prognosis upon cancer spread and growth, which results from the modulation of tumor dormancy, inflammatory response, and postoperative infection.^6–8^ Chemotherapy and radiotherapy exert notable influences on the immune system’s status, exhibiting pronounced toxicity toward normal tissue cells. Moreover, their efficacy in addressing deeply-rooted tumor tissues is somewhat restricted, thus presenting inherent challenges. Additionally, there exists a potential risk of developing drug resistance over time.^5,9^ As a result, these cytotoxic therapies often eliminate cancer cells at the expense of damaging normal tissues, resulting in unacceptable toxicity. Therefore, the objective of cancer treatment is to selectively eradicate tumors while minimizing harm to healthy cells. To achieve this, further research endeavors are required to develop effective targeted cancer treatment methods. In recent years, there has been significant progress in the field of immunotherapy, which has revolutionized traditional approaches to cancer treatment by shifting the focus from directly killing tumors to activating the host’s immune system. This helps the immune system fight against cancer and reduce off-target effects.^10^ However, the effectiveness of cancer immunotherapy in combating tumors is often hampered by various obstacles. These obstacles include limited tumor penetration, insufficient presence of tumor-infiltrating lymphocytes, and an immunosuppressive tumor microenvironment.^11^ Therefore, safe and targeted cancer therapies are in urgent need to overcome these limitations.

In recent years, the use of engineered bacteria has provided a unique treatment option to address these challenges. Compared with most other treatment methods, tumor-targeting engineered bacteria bear multiple pathways to inhibit cancer and have several advantages over other conventional therapies: The ability to target tumors specifically; Active proliferation in a variety of malignant tumors; Easy manipulation at the genetic level; Further programmed via sophisticated synthetic bioengineering to produce and deliver anticancer agents; Inexpensive production.^12,13^ More importantly, the remarkable capability of unlimited gene packaging enables recombinant bacteria to express and deliver a diverse range of therapeutic payloads, including chimeric toxins, cytokines, prodrug-converting enzymes, immunomodulators, and angiogenesis modulators for combating cancer.^14^ In addition, diversified engineering strategies have been applied into bacteria-based therapies, and these methods displayed competitive superiority for antitumor treatment.^13^ (Table 1) One case in point is that engineered bacteria can secrete antitumor substances such as bacterial toxins (*Staphylococcus aureus* alpha hemolysin) and lysozymes, thus directly attacking and killing tumor cells.^15,16^ Additionally, some bacteria can express prodrug-convering enzymes, a kind of molecule able to locally convert nontoxic prodrugs into drugs, such as *E. coli* cytosine deaminase (CD), which transforms the nontoxic prodrug 5-fluorocytosine into toxic 5-fluorouracil.^17^ Given the unique features of bacteria, like their intrinsic biocompatibility and motility, bacteria-based delivery systems have drawn wide interests in the diagnosis and treatment of various diseases, especially the cancer.^18^

**Table 1:** Engineering bacteria by chemical, biological and physical strategies

Engineering strategy	Bacteria	Therapeutic agents	PMID
Chemical Engineering	*E. coli*	Mesoporous silica nanoparticles loaded with doxorubicin.	33154882
	*Campylobacter jejuni*	Hyaluronic acid-decorated nanoparticle encapsulated Cytolethal distending toxin B	33557143
	*Salmonella typhimurium*	The conjugation of aptamers to bacterial surface	34782610
	*E. coli*	Cell-wall precursors	34822133
Biological Engineering	*Salmonella typhimurium VNP20009*	Anti-PD1 nanoantibodies	36213533
	*E.coli Nissle 1917*	Expressing a catalase plasmid containing the NSP4 signal peptide	33645010
	*E.coli*	Tumor necrosis factor-alpha (TNF-α)-neutralizing Nb	37003258
	*Salmonella typhimurium*	FlaB conjugated to hIL15 and mIL15	37148758
	*Escherichia coli Nissle 1917*	Increased intratumoural L-arginine concentrations	34616044
Physical engineering	*E. coli Nissle 1917*	αCTLA-4 and αPD-L1 nanobodies	35332124
	*E. coli Nissle 1917*	Lanthanide upconversion nanoparticles (UCNPs)	36314411
	*E. coli MG1655*	Induce Interferon-γ in an ultrasound-controllable manner	35918309
	*E. coli MG1655*	Cytolysin A (ClyA); Bi2S3 nanoparticles (BNPs) for sensitizing radiotherapy	35029367
	*E. coli Nissle 1917*	Facilitate ROS generation in response to X-ray irradiation	33645010
None-engineered	*Fusobacterium nucleatum*	Bacteria-self	34795206
	*Peptostreptococcus anaerobius*	Bacteria-self	34750535

*PD 1* programmed death 1, *NSP4* nonstructural protein 4, *hIL15* human interleukin-15, *mIL15* mouse interleukin-15, *UCNPs* upconversion nanoparticles, *ClyA* cytolysin A

**Fig. 1:**
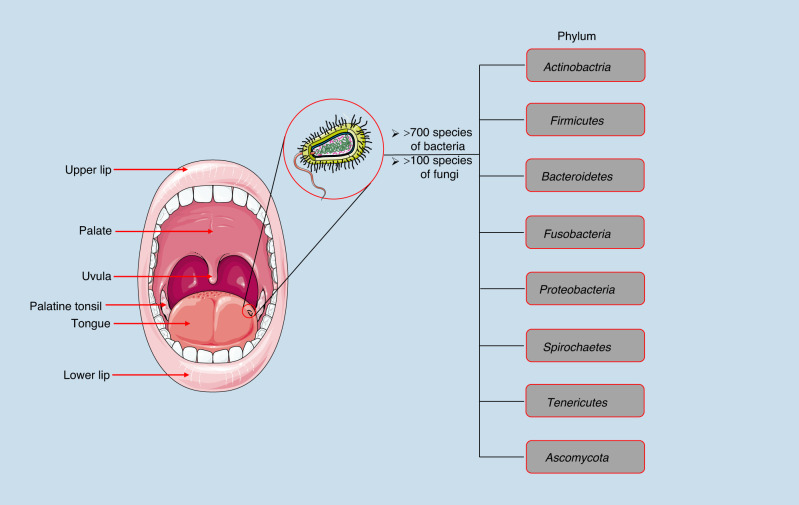
The diversity of oral flora. The human oral cavity is home to a diverse array of microorganisms, including over 700 species of bacteria and more than 100 types of fungi. These microorganisms are classified into eight different phyla: *Actinobacteria, Firmicutes, Bacteroidetes, Fusobacteria, Proteobacteria, Spirochaetes, Tenericutes* and *Ascomycota*

As the second largest microbiome habitat in human body, the human oral cavity harbors over 700 species of bacteria and over 100 types of fungi, with evidence of 296 species-level taxa in a typical individual’s mouth.^19,20^ (Fig. 1) Imbalance in the makeup of the oral microbiota is often caused by exposure to certain environmental factors, such as tobacco smoking, high sucrose intake, and antimicrobial use.^21^ The dysbiosis of the oral microbiota is associated with many systemic diseases, including oral squamous cell carcinoma (OSCC), colorectal cancer (CRC), pancreatic cancer (PC), Alzheimer’s disease and cardiovascular disease.^22–25^ Moreover, studies have shown that certain extensively researched periodontal organisms are now emerging as crucial factors in the evolving association between oral microbial dysbiosis and cancers,^26^ such as *Fusobacterium nucleatum* in colorectal cancer and esophageal cancer^27,28^ and *Porphyromonas gingivalis* in oral squamous cell carcinoma.^29^ With the development and refinement of 16S rRNA sequencing techniques, the study of the human microbiota has been greatly facilitated. Pioneering initiatives such as the Human Microbiome Project (HMP) have played a crucial role in unraveling the intricate composition of a healthy microbiome.^30^ Recently, the information concerning the composition of the oral microbiome and its impact on the development of cancer has rapidly evolved. However, the presence of exploitable engineered oral bacteria in such a diverse oral microbiome for effective cancer therapy is still rare in current research.

Engineering oral bacteria is a distinct category of microorganism that involve specific genetic editing or modifications of oral bacterial species. Engineering oral bacteria shares similar characteristics with general engineered bacteria, such as enhancing its activity by changing its genome or expressing specific genes, synthesizing specific molecules, or loading drugs and nanoparticles to achieve specific targeting effects.^31,32^ Moreover, it also retains the inherent features of oral microbes, such as: (1) Diversity: Oral microbiota exhibit a vast diversity of species and strains that can be harnessed and manipulated in engineering approaches. This diversity provides a rich pool of potential candidates for specific applications; (2) Biofilm Formation and Adhesion: Oral bacteria have the ability to form biofilms and adhesion to odontolith, which are structured communities embedded within a self-produced matrix. This capability enables engineered oral bacteria to colonize surfaces and enhance their persistence and functional efficacy; (3) Oral Environment Compatibility: Engineered oral bacteria are adapted to survive and thrive within the unique conditions of the oral cavity, including exposure to saliva, oral mucosa, and varying pH levels. This compatibility allows for better performance and longevity when used as therapeutic agents or carriers in the oral environment; (4) Microbial Interactions: Oral microbes engage in complex interactions and communication with other microorganisms in the oral ecosystem. By retaining these features, engineering oral bacteria can potentially exploit beneficial microbial interactions for enhanced therapeutic effects and colonization dynamics.^33–37^ Therefore, this review is based on the current research progress in oral microbiota and cancer, focusing on recent studies related to the use of engineered bacteria for cancer treatment and proposing new ideas for utilizing engineered oral bacteria in cancer therapy.

## Outline of the mechanisms of engineered bacteria targeting tumors

### Targeting and localization

Bacterial flagellar motors are composed of protein stators and rotors distributed along the bacterial flagella. These motors utilize the cellular chemical energy of bacteria to move in response to environmental cues, facilitating their ability to explore and adapt to different surroundings.^38–40^ These characteristics, such as regulation of pH value, nutrient concentration, oxygen content, light intensity, and drought wetness degree, are crucial in the design of drug delivery systems in engineering oral bacteria and guiding bacteria into specific locations.^41–43^ For instance, an attenuated *Salmonella* strain expressing Shiga toxin under the control of a promoter induced by low pH showed significant tumor selectivity and antitumor activity.^44^ Furthermore, the rapid proliferation of tumor cells led to the immature vascular structure inside the tumor center, which created a hypoxic tumor microenvironment (TME) and accompanied by the resulting metabolic disruption and immune cell suppression.^18,45–47^ However, such a microenvironment is considered attractive for the colonization and proliferation of anaerobic (such as *Clostridium and Bifidobacterium*) or facultative anaerobic bacteria (such as *Salmonella, Bacillus Subtilis, Listeria Monocytogenes* and *Escherichia Coli*).^48,49^ Besides, bacteria can also be genetically engineered to express binding peptides to selectively target cancer biomarkers and colonize tumors.^50,51^ Currently, the main method for evaluating the targeted characteristics of engineered bacteria is through luciferase reporter genes, such as *LuxCDABE* operon^52^ and *renilla luciferase* variant 8.^53^ Yet it is essential to note that the use of bacterial targeting for tumor therapy is in the early stages of research, and strategies to enhance the accuracy of bacterial localization, such as developing more specific molecular markers or optimizing the navigational abilities of the bacteria, still need more explorations.

### Therapeutic mechanisms

#### Direct and Indirect Cytotoxicity

Engineered bacterial strains exert their cytotoxic effects on tumor cells through various mechanisms. In one approach, the bacteria were modified to produce toxins that directly destroy tumor cells. For example, the *E. coli* strain K-12 was engineered to produce cytolysin A (ClyA) and treatment with *E. coli* expressing ClyA resulted in significant suppression of metastatic tumor growth and prolonged survival in mice model. Combination therapy with *E. coli*-expressing ClyA and radiation further enhanced tumor shrinkage and even led to complete tumor disappearance in a mouse model.^15^ In addition, the gene for *Staphylococcus aureus* α-hemolysin (SAH), a pore-forming protein, was cloned and inserted into *E. coli*, these engineered bacteria could penetrate effectively into tissue, cause cell death, and expand tumor necrosis.^16^ In addition to direct cytotoxicity, engineered bacteria could induce the release of antitumor cytokines.^54^ Cytokines constitute an extensive and diverse group of pro- and anti-inflammatory factors that play a crucial role in regulating host responses to infection, immune responses, inflammation, and trauma.^55^ IL-2 is the most widely studied cytokine in the context of bacterial delivery systems. Engineered *Salmonella Typhimurium* strains expressing truncated human IL-2 (SalpIL2) have demonstrated the ability to stably express IL-2. Treatment with these bacteria significantly inhibited the growth of osteosarcoma and pulmonary metastases.^56^ Furthermore, researchers have engineered *E. coli* DH5 to express tumor necrosis factor (TNF)-related apoptosis-inducing ligand (TRAIL), a molecule known for its potential in inducing cancer cell death and this approach holds promise for treating solid tumors.^57^ Overall, the use of engineered bacteria for targeted cytotoxicity and cytokine release represents a promising avenue for cancer treatment and holds potential for improving therapeutic outcomes.

#### Expression of prodrug‐converting enzymes

Recently, a novel approach for increasing the efficacy of bacterial therapy and reducing its therapeutic dosage is bacterial-directed enzyme prodrug therapy, which employs bacteria as enzyme carriers to convert a prodrug to a toxic drug specifically within the tumor site.^17^ For instance, researchers used *Escherichia coli* DH5α carrying the luxCDABE gene cluster and overexpressing β-glucuronidase for luminescent emission and enzyme expression, and studies found that cotreatment of 4T1, a highly metastatic mouse breast cancer cell line, in glycyrrhetinic acid and DH5α-lux/βG significantly decreased the IC50 values and delayed breast cancer growth in mouse model.^58^ In addition, Abigail et al.^59^ engineered a *E. coli* Nitroreductase NfsA to make an enzyme-prodrug activation system for improving the activity of therapeutically relevant prodrugs: the duocarmycin analog nitro-CBI-DEI, the dinitrobenzamide aziridine CB1954 and the 5-nitroimidazole metronidazole. They found that engineered bacteria can significantly improve prodrug-activating nitroreductase, which offers advantages for both targeted cellular ablation and suicide gene therapy applications.

#### Immune activation and immunomodulators

Engineering bacteria can indirectly induce innate or adaptive immune responses against tumor cells, such as *S. Typhimurium*^60,61^ and *Listeria. Monocytogenes* (*L. Monocytogenes*), *Clostridium. Novyi‐NT spores*.^62,63^ These artificial bacteria not only activate the immune system but also induce local tumor inflammation involving the significantly high expression of proinflammatory factors,^64^ such as TNF-α,^65^ IL-1β, IL-2, IL-12^66^ and IFN-γ.^67^ Zhang et al.^68^ engineered an attenuated strain of *Salmonella typhimurium*, which has the capability to secrete Vibrio vulnificus flagellin B (FlaB) conjugated with human or mouse interleukin-15 proteins. The administration of these bacteria to mice led to a remarkable alteration in the macrophage phenotype, transitioning from an M2-like state to an M1-like state. Furthermore, there was evident augmentation in the proliferation and activation of CD4+ T cells, CD8+ T cells, NK cells, and NKT cells within the tumor microenvironment. In recent years, advances in genetic technology and synthetic microbiology have allowed for the design of intelligent microbial delivery systems to enhance the therapeutic applications of immunomodulators.^69^ For instance, a nonpathogenic *E. coli* strain was successfully engineered to selectively release a CD47 nano-antagon within the tumor microenvironment, which resulted in accelerated tumor regression, heightened activation of tumor-infiltrating T cells, prevented metastasis, and long-term survival in a lymphoma mouse model.^70^ In addition, an engineered attenuated *Salmonella typhimurium* VNP20009 stably synthetizing IL-7 and granulocyte macrophage colony-stimulating factor (GM-SF) was found to recruit dendritic cells (DCs) and enhance T-cell priming to elicit an antitumor response.^71^ Similarly, studies indicated that engineered *S. typhimurium* overexpressing IFNγ could effectively suppress B16F10 melanoma tumor growth.^72^

#### Sensing of physical factors

Recently, the interaction between bacteria and external technologies outside of the tumor microenvironment has gained significant attention in research. Studies have demonstrated that engineering bacteria can be combined with materials and technologies such as ultrasound and magnetic-based approaches to enhance their behavior and optimize therapeutic strategies. It is known that ultrasound has numerous advantages in terms of noninvasiveness, safety, and tissue penetration.^73–75^ Because of the beam waves generated by ultrasound can be focused deep into the tissues, allowing for precise and localized elevation of temperature in the irradiated region, ultrasound provides an ideal remote modulation of gene expression when combining with temperature-based gene control elements.^76^ Recently, Chen et al.^77^ engineered an ultrasound-responsive bacterium (URB) capable of facilitating the controlled expression of exogenous genes in response to ultrasound stimuli. The hyperthermia induced by focused ultrasound exhibited a remarkable potential in promoting the expression of the IFN-γ gene, thereby enhancing the anti-tumor efficacy of the URB in tumor immunotherapy. In addition, the ultrasound-controllable engineered bacteria connecting their activity to the release of immune checkpoint inhibitors induced a marked suppression of tumor growth in clinical cancer immunotherapy.^78^ As for magnetic-based approaches, Akolpoglu et al.^79^ integrated magnetic nanoparticles and nano-liposomes loaded with photothermal agents and chemotherapeutic molecules onto *E. coli*. They found the engineered bacteria retained its original motility, able to colonize the tumor body under the drive of a magnetic field, and released drug molecules on demand through near-infrared stimulation. Furthermore, researches revealed a kind of magneto-aerotactic property of *Magnetococcus marinus* bacteria, enabling external magnetic torque-driven actuation. This unique property could be harnessed to enhance the engineering bacteria accumulation within the tumor microenvironment and facilitate the delivery of liposomal cargo.^80^

#### ROS production

**Fig. 2:**
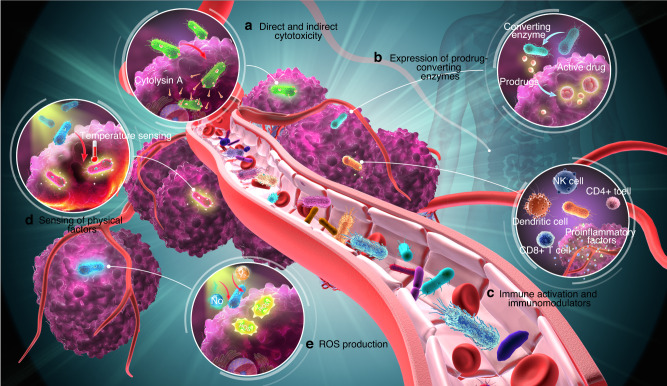
The Mechanisms of Engineering Bacteria Targeting Tumors. Advancements in nanomaterials and bioengineering technologies have led to the emergence of various killing mechanisms for tumor treatment using artificial engineered bacteria. Some of these mechanisms include: **a Direct and Indirect Cytotoxicity:** Engineered bacteria can be designed to produce and release cytotoxic molecules directly into tumor cells, leading to their death. Alternately, these bacteria can induce local immune responses that indirectly result in cytotoxic effects on tumors. **b Expression of Prodrug-Converting Enzymes:** Bacteria can be genetically modified to express enzymes that convert inactive prodrugs into active anti-cancer compounds specifically within the tumor microenvironment. **c Immune Activation and Immunomodulators:** Engineered bacteria can activate the immune system by releasing factors that attract immune cells or stimulate their function. Additionally, bacteria can be equipped with immunomodulatory proteins to enhance the body’s natural defense mechanisms against tumors. **d Sensing of Physical Factors:** Artificial bacteria can be engineered to sense unique physical characteristics, such temperature, magnetic field or hypoxia (low oxygen levels). Upon detection, these bacteria can trigger specific killing mechanisms, ensuring targeted destruction of cancerous cells while sparing healthy tissue. **e ROS Production:** Reactive oxygen species (ROS) are highly reactive molecules that can cause cellular damage. Engineered bacteria are being developed to generate ROS specifically within tumor cells, resulting in cell death through oxidative stress

Reactive Oxygen Species (ROS) are characterized as precarious oxygen-containing molecules. The prevalent ROS entities encompass singlet hydrogen peroxide (H2O2), oxygen (O2•), superoxide (•O2-), hydroxyl radical (•OH), nitric oxide (•NO), and peroxynitrite (ONOO-).^81,82^ ROS in cancer cells play a critical role in modulating and inducing apoptosis.^83^ Recently, the application of ROS in bacterial therapy is mainly focused on Photodynamic therapy (PDT), which utilizes photosensitizers (PSs) to produce reactive oxygen species (ROS) upon irradiation with the appropriate excitation light.^84^ Currently, PDT has been employed in the treatment of solid tumors and exhibited remarkable efficacy in eradicating tumor cells. For instance, Guo et al.^85^ have proposed the utilization of PDT-enhanced oncolytic bacterial immunotherapy (OBI) through the use of genetically modified *S. typhimurium* which express a fluorogen-activating protein (FAP). Upon stimulation of FAP, it leads to the generation of fluorescence and ROS so as to resulting in the killing of cancer cells and over-accumulated bacteria. Meanwhile, the destroyed bacteria and cancer cells can activate immune cells (such as macrophages, neutrophils, and dendritic cells), which release inflammatory cytokines (TNF-α and IL-1β) and reduce anti-inflammatory cytokines (IL-10), thereby further enhancing the efficacy of cancer immunotherapy. Mitochondria play a crucial role in anticancer strategies by targeting their metabolism (glycolysis and TCA cycle) as well as apoptotic and ROS homeostasis. It found that mitochondria-targeted cancer therapies in photodynamic therapy demonstrated superior effectiveness compared to non-targeting techniques with similar objectives.^86^ The mechanisms by which the aforementioned engineered bacteria targeting tumors are further illustrated in (Fig. 2).

## Application of engineered bacteria in tumor therapy

### Major administration routes

For the application of engineered bacteria in tumor treatment, it is known that the effectiveness of anticancer therapy much depends on the routes of administration. At present, major administration routes of engineered bacteria for cancer therapy including oral administration, intravenous administration, and intratumoral injection. Oral administration is widely regarded as the most convenient administration route, attributing to enhanced patient compliance and ease of use.^87^ However, when considering administration via oral route, it is imperative to take into consideration the impact of stomach acid and intestinal environment on the activity of the engineered microbiota as well as the release of anticancer drugs. At present, the engineered *Lactobacillus* and *Bifidobacterium* strains, known for their excellent acid stability properties, have been employed in attempts to develop oral vaccines aimed at tumor treatment.^88–90^

Intravenous administration of engineered bacteria is one of the primary administration routes in bacterial therapy and significant progress has been achieved in the field of in bacteria-mediated cancer therapy. For instance, in 2022, Liu et al.^63^ discovered that intravenous delivery of living *Listeria monocytogenes* induced tumor cell pyroptosis, effectively reversed the immunosuppressive tumor microenvironment, and stimulated a robust and long-lasting systemic anti-tumor immune response. This approach demonstrated outstanding therapeutic efficacy in treating solid tumors and inhibiting tumor metastasis. Similar results also found in *Bifidobacterium*,^91^ *Salmonella Typhimurium*,^92^ and *E. coli K-12*.^15^ Intravenous administration holds the potential to optimize the activity of engineered bacteria and enhance their anti-tumor effectiveness. However, it is accompanied by several concerns. One concern is how to prevent the uncontrolled proliferation of injected engineered bacteria within the bloodstream, which could lead to severe bacteremia. Another critical aspect involves in striking a delicate balance between the safe and effective dosage, ensuring that the engineered bacteria can accomplish their mission without causing significant harm to the body. These challenges still necessitate further exploration in the future.

The systemic biodistribution of engineered bacteria poses safety considerations that may necessitate the utilization of suboptimal dosages or even pose obstacles to their clinical advancement.^93^ Intratumoral injection offers the advantage of direct deposition of engineered bacteria within target tumors, effectively exerting a localized killing effect on the tumor cells. This approach brings about the distinct benefit of minimizing systemic toxicity compared to intravenous administration, as it has lesser impact on the body’s immune system. Gurbatri et al.^69^ reported an engineered probiotic bacterial system for the purposeful production and controlled intratumoral release of nanobodies that selectively target PD-L1 and CTLA-4. Their study showcased that a single injection of this engineered system significantly augmented therapeutic response, leading to tumor regression in mouse models. Moreover, studies found that intratumoral injection of genetically attenuated *Salmonella* coated with antigen-adsorbing cationic polymer nanoparticles resulted in enhanced activation of dendritic cells and systemic antitumor effects, and extended survival in multiple tumor models. Despite intratumoral injection of engineered bacteria offers notable advantages, this approach is not preferable for the treatment of deep-seated and vital organ related tumors.

### Immunotherapy

The role of engineered bacteria in anti-cancer immunotherapy has been documented for over one hundred years.^49^ As early as 1891, Dr. William B. Coley injected streptococcal organisms into a patient with inoperable cancer and successfully achieved the immunotherapeutic effect of tumor shrinkage. These bacteria or bacterial products were later called Coley toxins.^94,95^ The immune response caused by bacteria is taken seriously in the human body, particularly in local tumors.^96^ Due to the ability of obligate or facultative anaerobic microorganisms to infiltrate and proliferate in the hypoxic regions of tumors, bacteria-mediated tumor immunotherapy is becoming a promising cancer treatment method and has regaining attention to date.^97^ The application of engineered bacteria in promoting cellular immunotherapy is mainly through immune checkpoint inhibition, immune cell infiltration and tumor vaccines.^67,98^

The immune escape mechanisms employed by tumors encompass a wide range of immune checkpoint signaling pathways, notably involving programmed death-1 and its ligand (PD-1/PD-L1), CD47/signal regulatory protein-α (CD47/SIRP-α), and CD28/cytotoxic T lymphocyte-associated antigen-4 (CD28/CTLA-4).^99,100^ The activation of immune checkpoints can profoundly inhibit the recognition of immunocytes, thereby diminishing their ability to effectively eliminate tumor cells.^101^ In 2021, Zheng et al.^22^ reported that combination treatment with a nanoparticle incorporating hydrogel and exogenous *P. anaerobius* in murine OSCC tumors could synergize with checkpoint inhibition with PD-1. Moreover, the emergence of synthetic biology has facilitated the synthesis of diverse immune checkpoint inhibitors by using engineered bacteria. Examples include the production of nanobody antagonists targeting CD47 (*Escherichia coli*) and PD-1 (*Bifidobacterium*), which hold promise for enhancing immunotherapy approaches.^70,102^ Furthermore, Gurbatri et al.^69^ successfully engineered a probiotic bacterial system which could precisely and specifically target PD-L1 and cytotoxic T lymphocyte-associated protein-4 (CTLA-4). They employed a stabilized lysing release mechanism to enable controlled release of the nanobodies within the tumor microenvironment, and a single administration demonstrated profound efficacy in eliciting regression of tumors in syngeneic mouse models. Similarly, *Bifidobacterium pseudolongum*, *Lactobacillus johnsonii*, and *Olsenella species* also significantly enhanced the efficacy of immune checkpoint inhibitors in mouse models of cancer.^103^ In addition to their immune checkpoint inhibitor properties, tumor-targeting engineered bacteria have remarkable ability to facilitate the intratumoral infiltration of immune cells and evoke a potent inhibitory effect on tumor growth.^104–106^ For example, *Salmonella* bacteria have been engineered to secrete FlaB, a protein that can significantly induce the infiltration of immune cells, such as monocytes/macrophages and neutrophils.^54^ In terms of the application of bacterial immunotherapy, the classic example is the use of bacteria as vaccines. For instance, live bacteria such as *Bacillus Calmette-Guérin* (BCG) have been used clinically to fight the development of tumors.^107^ Inspired by this idea, diversified attenuated bacterial strains including *Listeria Monocytogenes*, *Salmonella Typhimurium*, and *Escherichia coli* were used as vaccines for cancer therapy.^108^

### Drug delivery systems

The emergence and development of materials science provide feasible ways and diversified options to obtain optimized engineered bacteria and realize artificial regulation for tumor treatment.^109^ For instance, the nanomaterials have now been integrated into therapeutic bacteria, such as *Salmonella*.^110–112^ In contrast to conventional drug delivery systems, bacteria and their extracellular vesicles (MVs) present unparalleled advantages as vehicles for drug delivery in cancer treatment.^113^ These unique entities have the capacity to effectively navigate through physical barriers, thus enabling precise targeting and accumulation within tumor tissues. Moreover, they have the remarkable ability to initiate potent antitumor immune responses, further enhancing their therapeutic potential in combating cancer.^114–116^

Recently, cell or bacterial constituents, including cell membranes, bacterial vesicles, and other active substances, have inherited their unique targeting properties and antitumor capabilities.^96^ Zhan et al.^117^ utilized a tetrahedral framework nucleic acid that was covalently conjugated with aptamer AS1411 and 5-fluorouracil (AT5). Subsequently, the oral pathogenic bacterium *Streptococcus mutans (S. mutans)* was employed as a biocarrier for synergistic biofilm targeting and immunomodulation. Subsequent investigations convincingly affirmed that these nanocells exhibited controlled release of elevated drug concentrations, accompanied by a profound immunomodulatory effect characterized by the potent induction of dendritic cell (DC) maturation and precise regulation of T cells. Furthermore, studies reported that the engineering of *Salmonella Typhimurium* bacteria with biotin molecules displayed on their outer membrane proteins were designed to bind to streptavidin-coated liposomes loaded with paclitaxel, resulting in enhanced antitumor efficacy compared to liposomes containing freely encapsulated drugs.^118^ *Escherichia coli* Nissle 1917 (EcN) is one of the best studied probiotic strains, and doxorubicin (DOX) was conjugated to EcN via acid-labile linkers of cis-aconitic anhydride (EcN-ca-Dox), thereby realizing the bacteria-directed accumulation and acid-responsive release of anticancer drugs in tumors.^119^ Moreover, *Escherichia coli* bacteria was engineered to adhere to drug-loaded polyelectrolyte multilayer (PEM) microparticles, which were intricately embedded with magnetic nanoparticles on their surface. This pioneering approach exemplifies the bacteria’s ability to efficiently transport doxorubicin to 4T1 breast cancer cells under precise magnetic guidance in vitro.^120^ In addition, multifunctional biohybrid microswimmers were fabricated by attachment of RBCs (loaded with doxorubicin and superparamagnetic iron oxide nanoparticles (SPIONs)) to bioengineered *Escherichia coli* MG1655 via a biotin-avidin-biotin binding complex, thus achieving the transformation from passive cargo carriers into active and guidable cargo carriers.^121^ Bacteria-based drug carriers possess distinctive attributes that enable in situ synthesis, release, and activation of pharmaceuticals in response to external stimuli. These remarkable characteristics offer significant advantages in alleviating the systemic toxicity of drugs to healthy tissues and impeding drug inactivation during transportation.^122^ Despite notable advancements in contemporary research pertaining to the development of anticancer drug delivery approaches, there also exist formidable impediments that demand immediate attention and resolution. These hurdles prominently involve in mitigating the cytotoxic impacts of therapeutic agents on normal cells, augmenting their targeted deposition within tumor sites, and amplifying their efficacy for eradicating malignant growth.

### Gene-targeted bacterial therapy

Encouraged by exponential advancements in genetic modification techniques and synthetic biology, the manipulation of bacteria through targeted gene deletions and the integration of multifaceted functionalities has become a reality. These deliberate modifications not only ensured the utmost safety of the bacteria but also bolstered the efficacy of tumor targeting by endowing the strains with an environmentally responsive nature.^111,123^ For instance, studies have demonstrated that lipopolysaccharides (LPS) found in the outer membrane of *Salmonella* contributing to the stimulation of tumor necrosis factor-alpha (TNF-α) production, thereby exhibiting a potent antitumor effect, while importantly, this factor can also contribute to severe inflammation or sepsis.^124^ Therefore, efforts were made to enhance the safety of *Salmonella* through LPS-related gene modification strategies, including targeted deletion of the virulence gene msbB. This genetic alteration leads to the loss of myristoylation of lipid A, thereby facilitating improved safety attributes.^125^ Moreover, Fan et al.^65^ constructed a noninvasive thermally sensitive programmable therapeutic system by using *E. coli* MG1655. This system involved in transforming the bacteria with plasmids expressing TNF-α and decorating them with biomineralized gold nanoparticles (AuNPs) to improve the effectiveness of tumor therapy.

Genetically engineered *Salmonella* can serve as a multifunctional platform for delivering customized payloads, such as DNA, RNA, or even proteins to tumor cells by using tools from synthetic biology and genetic engineering. For instance, the engineered strain of *S. Typhimurium*, known as JRG4401, has been specifically designed to deliver either a reporter gene (lacZ) or a novel therapeutic gene (HlyE) under the control of the FF + 20* promoter, which is highly sensitive and responsive to severe hypoxic conditions.^126^ In another representative example, attenuated *S. Typhimurium* ΔppGpp was integrated with an imaging reporter gene (Renilla luciferase variant 8 or lux) and transformed with the plasmid-encoded antitumor gene cytolysin A (ClyA) for targeted therapy against cancers.^53,127^ Moreover, to facilitate selective survival of *Salmonella* within the hypoxic tumor microenvironment while reducing toxicity to normal cells, an engineered strain named *Salmonella* YB1 has been derived by incorporating an essential gene under the control of a hypoxia-conditioned promoter.^128^ For RNA-related bacterial therapy, a study demonstrated the successful delivery of siRNA-PD-L1 using attenuated *Salmonella*, and the combination of lenvatinib effectively inhibited tumor growth and induced increased apoptosis in tumor cells.^129^ Furthermore, it was discovered that attenuated *S. Typhimurium* could effectively deliver shRNA-expressing vectors targeting STAT3 to hepatocellular carcinoma (HCC) cells and trigger RNA interference in vivo, leading to a substantial delay and reduction in HCC.^130^ Additional studies also shown the efficacy of using *Salmonella* as a drug delivery vehicle for administering proteins. An example highlighting the effectiveness of *Salmonella*-based bacterial delivery of constitutively active caspase-3 blocked the development of hepatocellular carcinoma and lung metastases.^131^ Moreover, the optimized attenuated *Salmonella Typhimurium* was utilized as a live vector to deliver antitumor molecules, including the angiogenesis inhibitor endostatin and apoptosis inducer TRAIL, and subsequent studies confirmed that the colonization of *Salmonella* bacteria in tumor tissue led to significant cell apoptosis and tumor angiogenesis suppression.^132^

### Clinical trial

**Fig. 3:**
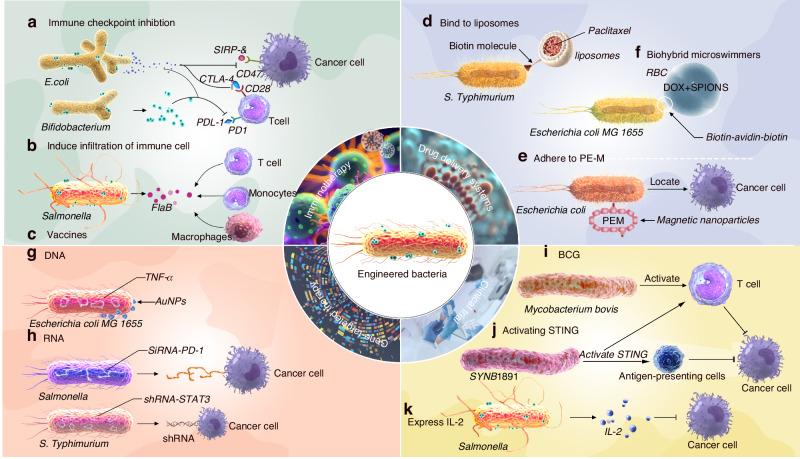
The application of engineered bacteria in tumor therapy. Engineered bacteria have gained prominencedue to their unique targeting advantages, which provide a wide range of applications in tumor treatment. Some of these applications include: **1**. **Immunotherapy**: Engineered bacteria can be employed as potent immunotherapeutic agents. **a** *E. coli* and *Bifidobacterium* can be engineered to express immune checkpoint inhibitors to enhance the effectiveness of immunotherapy. **b** Engineered *Salmonella* can express FlaB, ultimately inducing infiltration of immune cells. **c** Diversified attenuated bacterial strains were used as vaccines for cancer therapy. **2**. **Drug delivery systems**: bacteria can serve as efficient drug delivery vehicles. **d** *Salmonella Typhimurium* bacteria were designed to bind to streptavidin-coated liposomes loaded with paclitaxel to enhance antitumor efficacy. **e** *Escherichia coli* was engineered to embed with magnetic nanoparticles on their surface to achieve precise magnetic guidance drug delivery. **f** *Escherichia coli* MG1655 was attached RBCs (DOX and SPIONs) to achieve the transformation from passive cargo carriers into active and guidable cargo carriers. **3**. **Gene-targeted bacterial therapy**: engineered bacteria can be utilized for gene-targeted bacterial therapy. **g** TNF-α gene was introduced into *E. coli* MG1655 and decorated them with AuNPs to improve the effectiveness of tumor therapy. **h** Engineered bacteria can produce and deliver therapeutic molecules, such as siRNA or shRNA components, to target and modify cancer-related genes within tumor cells. **4**. **Clinical trials**: engineered bacteria are being evaluated in clinical trials for their safety and efficacy in tumor treatment. **i** BCG is the only US FDA-approved cancer bacteriotherapy that has been applied in the treatment of nonmuscle invasive bladder cancer. **j** The engineered bacterial strain SYNB1891 can activate the STING pathway in phagocytic APCs within tumors and it is currently undergoing evaluation in phase 1 clinical trial. **k** Engineered *Salmonella* stably expressing IL-2 has been tested in both canine and human clinical trials

Utilizing the pronounced antitumor advantages offered by bacteria, the field of bacteria-related microbes to fight cancer has experienced remarkable strides in both construction and application. These advancements have culminated in notable achievements, with selected outcomes now transitioning into rigorous clinical evaluation.^133^ For instance, the engineered bacterial strain, SYNB1891, is designed to specifically focus on activating the STING pathway in phagocytic antigen-presenting cells (APCs) within tumors and activates complementary innate immune pathways.^134^ SYNB1891 is currently undergoing evaluation in phase 1 clinical trial involving patients with advanced solid tumors and lymphomas (NCT04167137). Moreover, a live-attenuated *Listeria monocytogenes* was engineered to express mesothelin, a tumor-associated antigen highly expressed in Malignant pleural mesothelioma. Clinical trials confirmed that the synergistic administration of the engineered *Listeria monocytogenes* along with chemotherapy resulted in profound modifications within the local tumor microenvironment, yielding remarkable objective tumor responses in treated patients (NCT01675765).^135^ In addition, in clinical trials involving refractory cancer patients, the VNP20009 strain of *Salmonella typhimurium* have demonstrated a safe profile when administered to patients with some observable tumor colonization achieved at the highest tolerated dose. However, this trial also brought attention to the delicate balance between a safe and effective dosage because the highest tolerated dose was inadequate for achieving optimal tumor colonization, demonstrating the challenge of finding the right balance between safe dose and effective dose.^136^ Bacillus Calmette–Guerin (BCG), an attenuated strain of *Mycobacterium bovis*, is the only US Food and Drug Administration (FDA)-approved cancer bacteriotherapy that has been applied in the treatment of nonmuscle invasive bladder cancer.^137,138^ Additionally, the *Salmonella* strain expressing biologically active IL-2 was generated more than 20 years ago.^139^ IL-2-expressing *Salmonella* has been tested in both canine and human clinical trials.^140^ Different strains of *Clostridia, Lactococcus, Bifidobacteria, Shigella, Vibrio, Listeria, and Escherichia* have also been evaluated against cancer in animal models.^141^ The application of the aforementioned modified bacteria in tumor therapy is depicted in (Fig. 3).

## Overview of oral microbiota in carcinogenesis

**Fig. 4:**
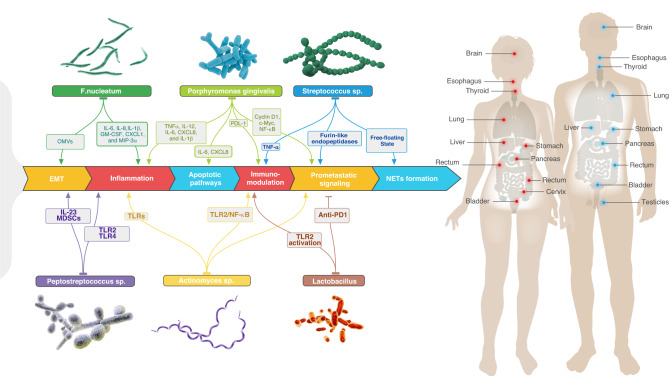
Oral flora affects the occurrence of multiple organ tumors. The oral microbiota plays a crucial role in the connection between oral health and the development of various organ tumors in the body. Several bacteria have been extensively studied for their potential impact on tumor occurrence and progression. These include *Fusobacterium sp, Porphyromonas gingivalis, Streptococcus sp, Peptostreptococcus sp, Actinomyces sp*, and *Lactobacillus*. The microbial communities within the oral cavity can influence tumor development through intricate signaling networks and mechanisms. Some of the key processes involved are: **(1). EMT (Epithelial-Mesenchymal Transition):** Certain oral bacteria have been found to induce EMT, a process that promotes the transformation of normal epithelial cells into more migratory and invasive mesenchymal-like cells. This transition is associated with enhanced tumor aggressiveness and metastasis. **(2). Inflammation:** Oral bacteria can trigger chronic inflammation in the oral cavity, which may contribute to an inflammatory microenvironment throughout the body. Chronic inflammation has been closely linked to the development and progression of various tumors. **(3). Apoptotic Pathways:** Bacterial products and components can disrupt normal cellular apoptosis pathways, which are responsible for programmed cell death. By manipulating apoptosis, these bacteria can potentially promote the survival and growth of tumor cells. **(4). Immunomodulation:** The presence of specific oral bacteria can trigger immune responses, leading to inflammation and immune cell recruitment. These immune responses can influence tumor development, either inhibit or promote tumor growth and progression. **(5). Prometastatic Signaling:** Some oral bacteria have been shown to enhance signaling pathways involved in tumor metastasis. Through these mechanisms, they can facilitate the spread of cancer cells to distant organs, contributing to the formation of secondary tumors. **(6). Formation of Nets:** Certain bacteria can influence the formation of neutrophil extracellular traps (NETs). These structures provide a protective environment where bacteria can survive and interact with host tissues, potentially promoting tumor development

The most extensively investigated relationship between engineering microbes and tumors focused on the gut microbiota, such as *Helicobacter pylori*,^142^ *Salmonella Typhimurium* strain^143^ and *Escherichia coli*.^144^ Nevertheless, despite being the second largest microbiome in the human body, limited research has been conducted on engineering the oral microbiome and its potential application in tumor therapy.^145,146^ A multitude of studies have reported significant correlations between oral flora and cancer. Moreover, our current understanding of the composition of the oral microbiome and its intricate influence on cancer is rapidly expanding. In the subsequent section, we delineate the associations between distinct members of the oral microbiota and cancer, and the relationships are likewise presented in (Fig. 4).

### *Fusobacterium sp.*

*Fusobacterium sp*. is an anaerobic, adhesive bacterium commonly found within the oral mucosa. It plays a significant role in biofilm formation and supports the establishment of a healthy oral microenvironment.^147^ However, *Fusobacterium nucleatum* (*F. nucleatum*) has also been associated with different types of cancers. For instance, studies have shown that the presence of *F. nucleatum* in tumor tissue is associated with poor overall survival in ESCC, early-stage HPV-negative tongue cancer, and increased metastasis in CRC.^148,149^ Studies have demonstrated notable distinctions in *F. nucleatum* profiles between patients with oral squamous cell carcinoma (OSCC) and healthy individuals, hinting at its potential as a biomarker for CRC.^150–153^ Furthermore, *F. nucleatum* has been identified in approximately 30% of breast tumors, as well as other breast cancer cohorts,^154,155^ and its abundance has been observed to increase in higher stages of breast tumors.^156^

The concrete oncogenic mechanisms of *F. nucleatum* in cancer are quite complex. For instance, hematogenous *F. nucleatum* could colonize CRC tissue using its lectin Fap2, which bond to tumor-expressed Gal-GalNAc, and the levels of Gal-GalNAc increased as human breast cancer progresses. The presence of *F. nucleatum* gDNA in breast cancer samples correlated with high Gal-GalNAc levels.^157^ Moreover, the OMVs secreted by *F. nucleatum* altered the expression levels of EMT-related proteins (E-cadherin, vimentin, and N-cadherin) and activated intracellular autophagy pathways, leading to the promotion of cancer cell migration and invasion.^158^ *F. nucleatum* could also influence the downstream oncogenic and prometastatic signalings. A recent study have shown that *F. nucleatum* both directly and indirectly modulate IL-8 and CXCL1 production in tumors, thus regulating cell seeding and metastatic potential, poor prognosis, and enhanced recruitment of tumor-associated macrophages and fibroblasts.^159^ Moreover, RNA sequencing and validation studies revealed that *F. nucleatum*-induced CRC lung metastasis was involved in the increased expression of the long non-coding RNAs keratin 7, antisense (KRT7-AS) and keratin 7 (KRT7) as well as the NF-κB signaling pathway.^160^ Caspase activation and recruitment domain 3 (CARD3), which is known for its role in inflammation and immunity, is a serine/threonine/tyrosine kinase with a carboxy-terminal caspase activation and recruitment domain (CARD).^161^ It found that infection with *F. nucleatum* could activate autophagy signaling by upregulating the expression of CARD3, thereby playing a crucial role in orchestrating CRC metastasis.^162^ In addition, *F. nucleatum* infection activated toll-like receptor 4 signaling (TLR4) to NF-κB and upregulated the expression level of microRNA-21 to increase the proliferation of colorectal cancer cells.^163^ A recent study have found that *F. nucleatum* can also stimulate neutrophil extracellular traps (NETs) formation via the TLR4-reactive oxygen species (ROS) signaling pathway and NOD-like receptor (NOD1/2)-dependent signaling, and that *F. nucleatum*-induced NETs indirectly accelerate tumor cell growth as well as tumor metastasis.^164^

Cancer chemoresistance is the result of complex interactions between gene regulation and the environment.^165^ Studies have shown that microbiota have the potential to modulate local immune responses, which in turn has an impact on chemotherapy outcomes.^166,167^ The current literatures suggested that *F. nucleatum* could modulate immune escape and inflammation within the tumor microenvironment.^168^ Specifically, the presence of *F. nucleatum* within human colonic tumors has been associated with the upregulation of inflammatory cytokines, such as IL-6, IL-8, IL-1*β*, GM-CSF, CXCL1, and MIP-3α.^169–172^ Moreover, *F. nucleatum* has the ability to target TLR4 and MYD88 innate immune signaling pathways, as well as microRNAs, to activate the autophagy pathway, thereby exerting regulatory control over the chemotherapeutic response in colorectal cancer.^173^ In addition, patients with metastatic CRC who failed to respond to immunotherapy had a greater abundance of *F. nucleatum* and increased succinic acid.^174^ *F. nucleatum*-derived succinic acid could inhibit the cGAS interferon-β pathway, consequently dampening the antitumor response by limiting CD8+ T-cell trafficking to the tumor microenvironment.^174^ It is known that programmed cell death protein 1 (PD-1), when bound to its ligand PD-L1, can inhibit T-cell activation and contribute to impaired antitumor immune responses.^175^ Recently, Gao et al.^176^ found that the presence of *F. nucleatum* was correlated with an improved therapeutic response to PD-1/PD-L1 blockade to modulate immune checkpoint therapy for patients with CRC. The potential interaction between *F. nucleatum* within cancer cells and immune checkpoint inhibitors is gaining significant momentum in future research.^177^ An *F. nucleatum*-specific bacteriophage, FNU1, was found to kill cells and significantly reduced the *F. nucleatum* biofilm mass. It has been recently suggested that FNU1 can be used to eradicate onco-bacterium from tumor tissue.^178^ Moreover, it found that antibiotic inoculation with *F. nucleatum* could eliminate *F. nucleatum* from breast cancer and further suppressed *F. nucleatum*-induced tumor growth.^157^ Although *Fusobacterium* has shown a strong potential therapeutic target for tumors, effective bacterial therapy based on *Fusobacterium* is currently limited and needs to be further explored in the future.

### *Porphyromonas gingivalis*

*Porphyromonas gingivalis* (*P. gingivalis*) is a Gram-negative oral anaerobe that is known to be involved in the pathogenesis of periodontitis.^179^ However, the role of this microbiota has been gaining increasing attention in the occurrence and development of certain cancers, particularly those of the oral cavity and orodigestive region. It has been reported that *P. gingivalis* infection is associated with orodigestive cancer, and the aberrant levels of serum anti–*P. gingivalis* IgG antibodies are found to be associated with cancer cell mortality, increased cancer cell invasion, and proliferation of cancer stem cells.^180,181^ Moreover, *P. gingivalis* was significantly abundant in esophageal cancerous tissue, and the salivary level of *P. gingivalis* was associated with the progression of ESCC.^182^ Additionally, a recent study highlighted the potential of *P. gingivalis* in saliva as a distinctive biomarker for the early detection of ESCC, and the ratios of *P. gingivalis*/*Prevotella* and *P. gingivalis*/All demonstrated notably enhanced diagnostic accuracy.^183,184^ In animal tumor-bearing experiments, samples infected with *P. gingivalis* in the tumor microenvironment exhibited the highest levels of cell invasion and proliferation, as well as the largest tumor volume.^185^ Similar results have also been observed in colorectal cancer,^186^ prostate cancer,^187^ and pancreatic cancer.^188^

The characteristics exhibited by *P. gingivalis* that possessing the potential to foster tumor development encompass the ability to produce carcinogenic metabolites, activate epithelial-to-mesenchymal transition, induce a dysbiotic inflammatory microenvironment, and impede apoptosis.^189^ For instance, phosphoethanolamine dihydroceramide (PEDHC) produced by *P. gingivalis* inhibits acid ceramidase expression, which caused intracellular ceramide accumulation and suppressed the survival and migration of OSCC cells in vitro.^190^ The process of epithelial-mesenchymal transition (EMT) entails sophisticated cellular metamorphosis, wherein epithelial cells undergo phenotypic alterations and adopt a migratory nature.^191^ Essential drivers of EMT are the transcription factors zinc-finger E-box-binding homeobox 1 and 2 proteins (ZEB1/2), which selectively bind to 5′-CACCTG sequences, exerting repressive effects on the transcription of epithelial-specific genes, such as E-cadherin.^192^ *P. gingivalis* displays a remarkable capacity to influence the activity of pathways governing epithelial-mesenchymal transition, eliciting an impressive degree of plasticity toward the mesenchymal phenotype.^193^ For instance, *P. gingivalis* strains devoid of the fimbrial protein demonstrated a reduced capacity to provoke ZEB1 expression. Targeted silencing of ZEB1 using siRNA effectively abrogated the *P. gingivalis*-induced upregulation of mesenchymal markers and inhibited epithelial cell migration.^194^ Moreover, *P. gingivalis* also exhibited the capacity to upregulate the expression of ZEB2, and this modulation was mediated through intricate pathways involving β-catenin and FOXO1.^193^

The delicate balance between pro- and anti-inflammatory responses elicited by bacteria is pivotal for the preservation of optimal health and homeostasis.^147,195^ *P. gingivalis* can foster the proliferation of the microbial community, precipitate dysbiosis, and subsequently disrupt the regulation of inflammatory responses.^196,197^ In particular, the presence of *P. gingivalis* elicited excessive secretion of proinflammatory cytokines, such as TNFα, IL-12, and IL-1β, culminating in consequent soft tissue degradation and progressive alveolar bone resorption.^147,197^ Furthermore, *P. gingivalis* possessed the capacity to elicit the secretion of interleukin-6 (IL-6) and orchestrated the production of CXC motif ligand 8 (CXCL8).^198^ IL-6 and CXCL8 demonstrated the potential to enhance the levels of matrix metalloproteinases, intensify cellular invasiveness, and exert modulatory influences on the expression of genes intricately involved in orchestrating cellular cycle dynamics and apoptotic pathways.^23,199^ Moreover, upon exposure to *P. gingivalis*, there was accelerated development within pancreatic cancer, and the microenvironment exhibited a preponderance of neutrophils, indicative of a proinflammatory state. Mechanistically, the intratumoral presence of *P. gingivalis* facilitated the progression of pancreatic cancer by amplifying the secretion of chemokines that attract neutrophils and inducing the release of neutrophil elastase (NE).^188^ The JAK/STAT pathway represents a swift membrane-to-nucleus signaling module capable of triggering the expression of multiple crucial regulators implicated in cancer and inflammation.^200^ Research has reported that the presence of *P. gingivalis* can activate JAK3, thus curtailing the production of IL-6 and TNF through ubiquitination-dependent Wnt3 degradation.^201^

The etiology and progression of *P. gingivalis*-induced cancer encompass a multitude of classical signaling pathways. For instance, *P. gingivalis* could orchestrate the upregulation of Cyclin D1 and c-Myc, the downstream targets of the NF-κB signaling pathway, thereby playing a role in inducing ESCC tumorigenesis and metastasis.^202^ Moreover, studies have indicated that the sonic hedgehog pathway and other cancer-related pathway genes are abnormally activated in the presence of *P. gingivalis* culture media.^203^ Gao et al.^204^ demonstrated that the fimbriae (FimA) of *P. gingivalis* exerted a potent influence on the expression of glycoprotein A repetition predominant (GARP), consequently triggering the activation of the TGFβ/Smad signaling cascade, thus fostering cancer cell proliferation and facilitating lung metastasis. Nod-like receptor (NLR) is a kind of innate immune receptor involved in the assembly of inflammasomes, and it participates in the innate immune response against pathogens such as *P. gingivalis*.^205^ A recent study showed that *P. gingivalis* promoted colorectal cancer through NLRP3 inflammasome activation and that the effect of NLRP3 on *P. gingivalis* pathogenesis was mediated by hematopoietic sources.^206^ Grainy head-like 3 (Grhl3) is a member of a highly conserved family of transcription factors which is critical for epidermal development and homeostasis.^207,208^ It has been discovered that infection with *P. gingivalis* elicits downregulation of GRHL3 and PTEN while concurrently upregulating p-Akt levels within esophageal cancer cells, thereby accentuating the proliferation and migratory potential of ESCC cells.^209^ Moreover, *P. gingivalis* instigated a substantial elevation in the expression of the GSK3β protein within ESCC cells, consequently propelling the advancement and development of chemoresistance via GSK3β-mediated mitochondrial oxidative phosphorylation (mtOXPHOS) in human ESCC.^210^ Guo et al.^185^ indicated that *P. gingivalis* could recruit TANs via activation of the CXCL2/CXCR2 axis in the TME of OSCC, concurrently activating the JAK1/STAT3 signaling pathway and epithelial-mesenchymal transition, thereby promoting OSCC progression. Bacterial infection has the capacity to induce alterations in PD-L1 expression, and viable or heat-killed *P. gingivalis*, along with the membranes of *P. gingivalis*, have demonstrated the ability to induce robust PD-L1 expression in cancer cells.^211^ For instance, PD-L1 was demonstrated to be upregulated in prostate cancer cells after infection with *P. gingivalis* membrane fractions by the NOD1/NOD2 signaling pathway.^187^ Moreover, it found that *P. gingivalis* infection elicited the elevation of PD-L1 expression on dendritic cells (DCs) through the Akt-STAT3 signaling pathway, thereby dampening CD8+ T-cell cytotoxicity and exacerbating the growth of oral cancer cells.^212^ Additionally, *P. gingivalis* could inhibit PDCD4 (programmed cell death factor 4) expression and lead to cancer stem cells (CSCs) enrichment in ESCC cells. After *P. gingivalis* elimination, PDCD4 expression was upregulated, and the percentage of CSCs, chemoresistance and malignancy were decreased in ESCC.^213^

### *Streptococcus sp.*

The relationship between certain *streptococci* and carcinogenesis has been known for many years.^214^ For instance, Coley’s toxins, an early form of cancer immunotherapy, were based on *Streptococcus pyogenes* (*S*. *pyogenes*).^215^ In recent studies, the metabolites of *S. pyogenes* were examined, revealed that *S. pyogenes* acted as an agonist of the TLR2-TLR1 signaling pathway, displaying a 6 μM EC50 (median effect concentration). This stimulation led to a robust induction of TNF-α, signifying potential implications for immune regulation and cancer immunotherapy.^216^ Research has shown that there is a close association between *Streptococcus* and the occurrence and development of head and neck cancer.^214,217^ A recent study found that *S. anginosus* is elevated in patients with OSCC, highlighting the continued relevance of this bacterium in the carcinogenesis of the oral cavity.^218^ Oropharyngeal cancer and cervical cancer are known to be associated with human papillomavirus (HPV) infection.^219,220^ Studies have found that oral *Streptococcus* can produce furin-like endopeptidases, which potentially influence HPV tissue tropism and contribute to the occurrence of carcinogenesis in the oral cavity and throat.^221^ Moreover, the proliferation of *Streptococcus* was observed to exhibit a direct positive association with the infiltration of GrzB+ and CD8+ T cells within tumor tissues, and the abundance of *Streptococcus* effectively forecasted an extended period of disease-free survival in patients diagnosed with ESCC.^222^ As one of the main components of dental plaque, *Streptococcus mutans* is usually thought to be a pathogenic bacterium of dental caries.^223^ Recently, it was found the presence of *Streptococcus mutans* infection was associated with epithelial-mesenchymal transition, increased tumor aggressiveness, and interleukin-6 (IL-6) production in OSCC.^224^ Furthermore, research has revealed that *Streptococcus mutans* possesses the ability to facilitate the metastasis of breast cancer cells to the lungs through vascular inflammation and disruption of vascular barrier function.^225^ Additionally, *Streptococcus* can serve as a microbial marker for the occurrence and metastasis of oropharyngeal and pancreatic cancers.^226,227^ The involvement of *Streptococcus* in tumor development is also intricately linked to several classical signaling pathways. For instance, the lower airways of patients with lung cancer were found to be enriched for *Streptococcus*, which was associated with upregulation of the ERK and PI3K signaling pathways.^228^ Moreover, *Streptococcus* exhibited a compelling correlation with the NOD/RIP2/NF-κB signaling cascade, and this intricate interplay could be modulated by several chemotherapeutic agents.^229^ Baraniya et al.^230^ found the transcriptional alterations caused by *Streptococcus mitis* primarily exhibited an anticancer effect, marked by the inhibition of the HOTAIR regulatory pathway, JAK/STAT signaling, Cyclin/Cyclin-dependent kinases, and endothelin1 signaling pathways. Neutrophil extracellular traps (NETs), intricate lattice-like structures composed of DNA-histone complexes and proteins discharged by activated neutrophils, were initially identified for their essential involvement in antimicrobial resistance and immune regulation.^231–233^ The present study revealed that the formation of neutrophil extracellular traps (NETs) was differentially influenced by *S. mutans* biofilms and their planktonic counterparts. The free-floating planktonic *S. mutans* exhibited a vigorous and proactive NETs response, whereas the biofilm community displayed a strikingly contrasting negative NETs response.^234^ Additionally, the oral pathogenic bacterium *S. mutans* was also employed as a biocarrier for synergetic biofilm targeting and immunomodulation.^117^ At present, bacterial-based cancer therapies involving *Streptococcus* are relatively rare, and more clinical studies are required to verify this effect.

### *Peptostreptococcus sp.*

*Peptostreptococcus* is a Gram-positive bacterium that is part of the normal bacterial flora found in various parts of the body, including the oral cavity, intestines, and urogenital tract. *Peptostreptococcus* species are known for their ability to thrive in anaerobic (oxygen-depleted) environments. In tumor microenvironment, where low oxygen levels are commonly present due to inadequate blood supply, these bacteria can flourish.^45,46^ *Peptostreptococcus* has long been implicated as a causative agent of several diseases, including endocarditis and infections of the genitourinary and gastrointestinal tracts.^235–237^ In recent years, increasing evidence has elucidated the intricate association between *Peptostreptococcus anaerobius* (*P. anaerobius*) and the pathogenesis of diverse malignancies, such as colorectal cancer, oral squamous cell carcinoma, and gastric cancer. For instance, recent investigations revealed that there were conspicuous enrichment of *P. anaerobius* within the intestinal microbiota of patients suffering from chemoresistant colorectal cancer (CRC), and the abundance of *Peptostreptococcus* was indicated to be a potential microbial marker for the risk prediction of colorectal neoplasia.^173,238–242^ Moreover, *P. anaerobius* has the potential to directly educate CRC cells and their microenvironment, contributing to the promotion of cancer progression.^243^ Except for colorectal cancer, after performing 16 S rRNA amplicon sequencing on 54 oral swab samples from OSCC patients, researchers found *Peptostreptococcus* was significantly enriched in OSCC recurrence, and it demonstrated a high diagnostic power or microbial marker for OSCC metastasis.^22,244–246^ Furthermore, a meta-analysis has uncovered the augmented presence of the opportunistic pathobiont *Peptostreptococcus* in gastric cancer, with its abundance increasing progressively during the cancer progression.^247^ Existing microbial data based on 16 S rRNA analysis have demonstrated substantial variations in *Peptostreptococcus* levels between gastric and nongastric cancer cases, showcasing its potential diagnostic efficacy and broad applicability for patients afflicted with gastric cancer.^248^

The intricate carcinogenesis mechanism attributed to *Peptostreptococcus* is little- known. It found that *P. anaerobius* could upregulate genes responsible for AMP-activated protein kinase (AMPK) signaling and Toll-like receptor (TLR) signaling, thus enhancing the proliferation of colon cancer cells.^243^ The interaction of *P. anaerobius* with TLR2 and TLR4 expressed in colon tissues led to an increase in cholesterol synthesis and cell proliferation, reactive oxidative species (ROS) levels and a pro-inflammatory response.^240,249^ In addition, *P. anaerobius* can recruit myeloid-derived suppressor cells (MDSCs) into the tumor microenvironment. The increased secretion of IL-23 by MDSCs can activate the STAT3-EMT pathway, subsequently facilitating the epithelial-mesenchymal transition (EMT) of tumor cells to induce chemoresistance in CRC.^250^

### *Actinomyces sp.*

The *Actinomyces* genus consists of filamentous, nonspore-forming, Gram-positive bacilli. These bacteria are predominantly facultative anaerobes and are primarily known for their infrequent but significant contribution to abscess formation.^251^ However, *Actinomyces* infection has also been noted to simulate symptoms commonly associated with malignancy. Recent research uncovered *Actinomyces* as a noteworthy constituent of the microbiota in young-onset colorectal cancer (yCRC). Remarkably, *Actinomyces* were found to colocalize with cancer-associated fibroblasts within the yCRC, and this symbiotic interaction activates the TLR2/NF-κB pathway, ultimately resulting in a dampened influx of CD8+ T lymphocytes into the CRC microenvironment.^252^ Moreover, 16S rRNA amplicon sequencing from OSCC patients showed that *Actinomyces* was significantly enriched in the recurrence of OSCC.^244^ The abundance of *Actinomyces* was found to be significantly decreased after surgical operation in patients with NSCLC (non-small cell lung cancer).^253^ Moreover, oral *Actinomyces* interacted strongly with methylation changes in immune genes, which was associated with patient prognosis.^254^ Chua et al.^255^ conducted a study demonstrating that the increased abundance of *Actinobacteria* in the gut was associated with heightened immune activation among survivors of acute lymphoblastic leukemia (ALL) who experienced chronic inflammation. Currently, there is a limited amount of research on the relationships between *actinomycetes* and cancer. However, an emerging pattern suggests an escalating interest in this field, underscoring the necessity for in-depth exploration in forthcoming investigations.

### *Lactobacillus sp.*

*Lactobacillus* is a kind of probiotic found in the oral cavity and other internal organs. *Lactobacillus reuteri* can be used as drug carriers for surface-encapsulated mesoporous nanoparticles, and this bacterioboat showcased exceptional drug-loading capacity, achieving a remarkable drug payload of up to 16% relative to its dry weight. Moreover, it exhibited precise intestinal anchorage primarily concentrated within the alveolar zones, thereby facilitating targeted drug localization and delivery.^256^ Besides, it found that oral administration of a probiotic *Lactobacillus. salivarius* REN or its secretions demonstrated significant efficacy in suppressing 4NQO-induced oral carcinogenesis, both in the initial and post-initial stages, and this inhibition showed a dose-dependent relationship.^257^ Gao et al.^258^ confirmed that the administration of *Lactobacillus rhamnosus* Probio-M9 significantly improved tumor inhibition by enhancing the efficacy and responsiveness of anti-PD-1-based immunotherapy. Exopolysaccharides (EPS) derived from *Lactobacillus* have been shown to play crucial roles in anti-cancer, immunomodulatory and anti-viral activities.^259^ Studies have identified that the EPS derived from *Lactobacillus acidophilus* ATCC 4356 exhibit significant efficacy in controlling hepatocellular carcinoma development. This effect is believed to be mediated through the regulation of the TLR2/STAT-3/P38-MAPK pathway.^260^ This finding highlights the potential of EPS from *Lactobacillus* as a promising therapeutic intervention for cancer.

## Future expectations

Infectious agents are known to contribute to the development of cancer, with 15% of cancer cases being associated with a specific pathogenic microorganism.^261^ In recent years, significant advancements in bioengineering technology and nanomaterials have generated numerous reports on the utilization of engineered bacteria for cancer treatment. However, the majority of these reports have primarily centered around digestive tract microorganisms, leaving a dearth of research focused on engineered oral bacteria, although the oral cavity represents the second largest microbial reservoir in the human body. Compared to the microenvironment in the digestive tract, the oral microbiota exists in a milder environment with lower pathogenicity. Furthermore, due to the presence of saliva, there is a certain balance and interaction between the oral and digestive tract microbiota. Therefore, the oral microbiota may be more suitable for engineering applications to treat other diseases. One characteristic of the oral microbiota is its diversity and complexity. The oral cavity harbors one of the most abundant microbial habitats in the human body, with billions to trillions of microbial cells per millilitre of saliva, encompassing a wide array of species.^262^ Moreover, the oral cavity is divided into four distinct microhabitats, each harboring a unique microbiota composition and serving specific functions in maintaining oral health.^30,145^ This high level of diversity implies the existence of potential metabolic capacities and functionality within the oral microbiota that can be harnessed to address various diseases.

Another characteristic lies in the reciprocal influence between the oral microbiota and the gastrointestinal microbiota.^146^ It is worth noting that there is a physical as well as chemical connection between the oral cavity and gut.^263^ Through dissemination via saliva, oral microbes can enter the digestive tract and interact with the gastrointestinal microbiota. For instance, *Bifidobacterium* stands as the prevailing bacterial genus within the neonatal gut, however, the gut-dwelling *Bifidobacterium* has been detected in the oral fluid, unveiling a particular association between these two seemingly distinct environments.^264,265^ Moreover, among the elderly adults, there is a discernible prevalence of oral bacteria migrating to the gut in contrast to their healthy adult counterparts, such as *Porphyromonas, Fusobacterium*, and *Pseudoramibacter*.^266,267^ This exchange and interaction can lead to the formation of symbiotic relationships, contributing to the regulation of the overall microbial community balance.^268^ The Oral-Gut microbiome axis also received considerable attention with regards to its pivotal role in the progression of cancer. Examples include *Parvimonas*, *Peptostreptococcus*, and *Fusobacterium*, which have been identified in the gut microbiota of patients afflicted with colorectal cancer.^269^ Moreover, it is well established that dysbiosis in the gut microbiome can significantly impact the progression of hepatocellular carcinoma (HCC)^270^. Interestingly, HCC patients exhibit a notable abundance of the *Haemophilus*, *Porphyromonas*, and *Filifactor* genera within their salivary microbiota and *Oribacterium* and *Fusobacterium* on the tongue coat, suggesting the pivotal role of Oral–Gut microbiome axis in the progression of HCC.^271,272^ However, the majority of researches concerning the oral and gut microbiomes has been conducted separately in an organ-specific manner, rather than adopting a comprehensive integrative approach.^273^ Therefore, the utilization of engineered oral microbiota exhibits the potential to function as an interlinking agent connecting the oral cavity and the gastrointestinal tract. Considering the interconnection between oral microbiota and gastrointestinal microbiota, this strategy can potentially be extended to address a broader spectrum of gastrointestinal-related diseases.

At present, the application of engineered oral bacteria for tumor treatment is still in the early stage, and only a few specific microbes have been utilized for this purpose. Examples include *Bifidobacterium, Lactobacillus* and *P. anaerobius*. For instance, Kikuchi et al.^274^ genetically modified *Bifidobacterium* to express and secrete the trastuzumab single-chain variable fragment (scFv). This recombinant scFv successfully bound to the cell surface HER2 receptors and demonstrated the ability to inhibit the growth of HER2-positive cancer cells. Moreover, recombinant *Bifidobacterium* displaying Wilms’ tumor 1 protein was used to develop oral cancer vaccine.^275^ Additionally, reduced oxygen environments create an ideal habitat for the colonization of *Bifidobacterium*.^276^ Tang et al.^277^ elaborately engineered an AP-PFH/PLGA nanoparticles (NPs) system that possessing the ability to specifically target *Bifidobacterium bifidum* (BF) colonized tumors. This targeted delivery approach significantly enhanced the efficacy of high-intensity focused ultrasound (HIFU) therapy for cancer treatment. As a widely recognized probiotic bacteria, *Lactobacillus* is being actively engineered and harnessed as a potential therapeutic tool for combating tumors. Espinal et al. engineered the second-generation probiotic *Lactobacillus*-*reuteri*-Interleukin-22 (LR-IL-22), it was found LR-IL-22 has shown remarkable improvement in multiple biomarkers associated with radiation damage to the intestine, immune system, and bone marrow, which can facilitate therapeutic whole-abdomen irradiation for widespread intra-abdominal ovarian cancer.^278,279^ Besides, an innovative bacterial drug delivery system has been developed using the lactic acid bacterium *Pediococcus pentosaceus*. This engineered bacterium is equipped with dual gene cassettes driven by a strong inducible promoter, encoding the therapeutic protein P8 fused to a secretion signal peptide and a complementation system. Subsequent research found that this engineered probiotic significantly reduced tumor volume and inhibited tumor growth in CRC mouse model.^280^ Studies have revealed that certain biomaterials can exert anti-tumor effects through *P. anaerobius* mediation. A significant finding suggested that adhesive hydrogel incorporating silver nanoparticles could regulate *P. anaerobius* homeostasis to synergizes with PD-1 blockade in mice with oral squamous cell carcinoma.^22^

Despite the myriad advantages offered by engineered bacterial therapy, which conventional therapies fail to provide, it remains constrained by a multitude of limitations that necessitate swift innovation and resolution. First, the therapeutic utilization of bacteria-related microbes necessitates stringent adherence to the regulations and guidelines established by regulatory authorities, notably the US Food and Drug Administration (FDA). This adherence is imperative to assure both the robust efficacy of the drug and the unwavering safety of patients undergoing treatment. Conforming to these regulatory frameworks ensures that meticulous protocols are followed throughout the arduous stages of development, rigorous testing, and ultimate approval of bacteria-based therapies. In doing so, it guarantees that the requisite benchmarks of quality, safety, and efficacy are met prior to the introduction of these treatments into clinical practice.

The second concern is the potential risk of autoimmune diseases arising from the introduction of live biotherapeutics into the body, and bacterial toxicity may induce adverse reactions or harmful effects in the host. As a result, thorough screening and selection of nonpathogenic or attenuated strains are crucial steps in mitigating this risk and maintaining patient well-being. Moreover, the potential for gene mutations within the administered bacteria represents another significant hurdle. Genetic changes can alter the intended therapeutic properties of the bacteria or, in some cases, render them ineffective altogether. Consequently, it is imperative to address this challenge through robust strategies aimed at preserving the genetic stability of the bacterial strains employed. Ensuring that the engineered bacteria retain their intended functionality throughout treatment becomes a critical consideration, and developing strategies to maintain genetic stability within the administered bacterial populations is vital for consistent and predictable treatment outcomes. Finally, uncontrollable proliferation of bacteria within the body following administration is a common and tricky problem. Bacterial overgrowth may lead to unanticipated consequences, such as an imbalance in the host’s microbiota or the escalation of adverse effects. The development of stringent control mechanisms to regulate bacterial growth and prevent uncontrolled proliferation is of paramount importance in ensuring the safety and therapeutic efficacy of these interventions. While there are still challenges and limitations in utilizing the oral microbiota for engineering interventions, this area of research presents novel opportunities for discovering innovative cancer treatments. By delving into the intricacies of the oral microbiota and its relationship with overall human health, we have the potential to pave the way for more effective prevention and treatment approaches for cancer treatment in the future.
